# The Association of Serum Bisphenol A with Thyroid Autoimmunity

**DOI:** 10.3390/ijerph13111153

**Published:** 2016-11-17

**Authors:** La-or Chailurkit, Wichai Aekplakorn, Boonsong Ongphiphadhanakul

**Affiliations:** 1 Department of Medicine, Ramathibodi Hospital, Mahidol University, Bangkok 10400, Thailand; boonsong.ong@mahidol.ac.th; 2 Department of Community Medicine, Ramathibodi Hospital, Mahidol University, Bangkok 10400, Thailand; wichai.aek@mahidol.ac.th

**Keywords:** bisphenol A, antithyroglobulin antibody, antithyroperoxidase antibody, antithyrotrophin receptor antibody, autoimmunity

## Abstract

*Introduction*: Data on the association of bisphenol A (BPA) exposure and autoimmunity in humans is unclear. *Objective*: To elucidate the influence of BPA on thyroid autoimmunity, in the present study we assessed the association between serum BPA and thyroid autoantibodies. *Methods*: Serum samples from 2361 subjects, aged ≥15 years, from the Thai 4th National Health Examination Survey were measured for BPA, antithyroglobulin (TgAb), antithyroperoxidase (TPOAb) and antithyrotrophin receptor (TRAb) antibodies. *Results*: The proportion of subjects positive for TgAb, TPOAb and TRAb were 11.1%, 14.9% and 1.9%, respectively. With regard to BPA, 51.9% had serum BPA levels exceeding the detection limit of the assay (0.3). There was a significant increasing trend for subjects with TgAb (*p* < 0.05) and TPOAb (*p* < 0.001) positivity as BPA quartiles increased, particularly in the highest quartile. In contrast, no relationship between BPA quartiles and TRAb was found. Logistic regression analysis showed that age, gender and BPA quartiles were determinants of TPOAb or TgAb positivity, independent of BMI. However, only the association between BPA and TPOAb positivity was consistent in both men and women. *Conclusions*: BPA was independently associated with TPOAb positivity. However, its mechanism related to TPOAb positivity, subsequently leading to autoimmune thyroid disease, needs further investigation.

## 1. Introduction

Bisphenol A (BPA), the monomeric form of polycarbonate plastic, is an endocrine disruptor that has been implicated in a number of disorders. BPA can affect autoimmunity both directly and indirectly [1]. For example, it is well established that estrogen enhances autoimmunity; BPA, by acting as a xenoestrogen, can influence autoimmunity similarly to estrogen [2]. Moreover, BPA can increase serum prolactin, which may have a direct enhancing effect on autoimmunity [3]. The role of BPA in disrupting cytochrome P450 and stimulating the production of reactive oxygen species has also been suggested [4].

Although the effects of BPA on autoimmunity in experimental animals and cell culture systems appear to be more or less consistent, the association of BPA exposure and autoimmunity in humans is much less clear. In the U.S. National Health and Nutrition Examination Survey (NHANES), BPA and triclosan were shown to be likely to negatively affect human immune function, as assessed by serum cytomegalovirus antibody and allergy or hay fever diagnosis [5]. We previously reported a negative association between serum BPA and serum thyroxine (T4) in males with positive serum thyroid antibodies [6] which is in agreement with a recent study in U.S. adults which suggested an inverse relationship between urinary BPA and total T4 [7]. Moreover, we have also demonstrated that estradiol is associated with thyroid autoimmunity in males [8]. The three most important antigens in autoimmune thyroid disease are thyroglobulin, thyroid peroxidase and TSH receptor. The prevalence of antibodies to these antigens varies according to the type of the autoimmune thyroid disease. It is unclear at present how BPA might differentially affect these thyroid antibodies. To further elucidate the influence of BPA on thyroid autoimmunity, we assessed the association between serum BPA and thyroid autoantibodies in the Thai 4th National Health Examination Survey (NHES IV) cohort.

## 2. Methods

This study used data and a subsample of sera from the 4th National Health Examination Survey, a representative cross-sectional survey of the Thai population. The survey was conducted in 2009 and included a total sample size of 21,960 Thais aged 15 years and over. Details of the sampling method have been described elsewhere [9]. In brief, the participants were randomly selected from 21 provinces in four regions of Thailand and the city of Bangkok. The present study drew a subsample of the data by dividing the total sample into 24 strata based on sex, area of residence (urban/rural for each of the four geographic regions and Bangkok, which was regarded as an urban area only) and age-specific groups (15–29, 30–44, 45–59, 60–69, 70–79 and ≥80 years of age). In each stratum, 25 individuals were randomly selected from each region and Bangkok; ultimately, a total of 2700 Thais were sampled. The study was approved by the ethics committee of Ramathibodi Hospital (Project Identification Code: MURA2016/485). Informed consent was obtained from all subjects.

### 2.1. Data Collection

Data collection included a face-to-face interview conducted in the community and a subsequent health examination and blood sample collection. Weight and height were measured by trained field staff using standard procedures. Venous blood samples were obtained from participants in the morning after fasting overnight. Serum samples were transferred and stored at −80 °C.

### 2.2. Serum Analysis

Serum BPA levels were determined by competitive ELISA (IBL International, Hamburg, Germany) with a detection limit of 0.3 ng/mL. Intra- and inter-assay precision were 7.0% and 13.6%, respectively. Serum thyroglobulin antibody (TgAb), thyroid peroxidase antibody (TPOAb) and thyroid receptor antibody (TRAb) levels were measured by electrochemiluminescence immunoassay on a Cobas e 411 analyzer (Roche Diagnostics, Mannheim, Germany). The assays had intra-assay precision of 6.1%, 9.2% and 11.5%, respectively. In the present study, levels of TgAb, TPOAb and TRAb above 115 IU/mL, 34 IU/mL and 1.75 IU/L, respectively, were considered positive.

### 2.3. Statistical Analysis

After excluding those with serum thyroid-stimulating hormone (TSH) levels lower or higher than 0.34 and 5.11 IU/mL, respectively [10]. On the basis of a likelihood of thyroid disorder, a total of 2361 samples were available for analysis. Serum BPA was categorized into quartiles (≤0.071, 0.072–0.317, 0.0318–0.749 and ≥0.750 ng/mL). Discrete data were reported as percentages and compared using a chi-squared test. All continuous data in this study were not normally distributed. Therefore, the continuous data were reported as median and range and analyzed by Mann-Whitney *U*-test for comparison of two groups. Multivariate analyses were performed using a logistic regression model to identify independent predictors of positive thyroid autoantibodies. Statistical significance was established at *p* < 0.05. All statistical analyses were performed using SPSS for Windows version 20.0 (SPSS Inc., Chicago, IL, USA).

## 3. Results

**Figure 1:**
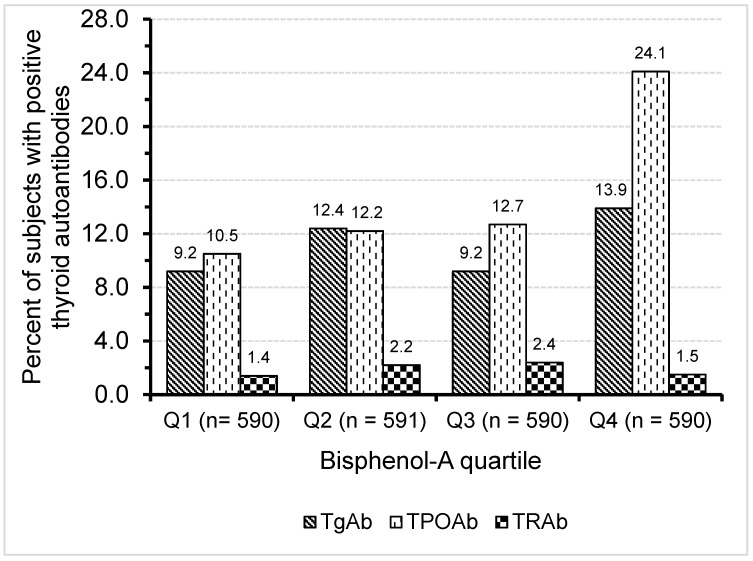
Percent positive rate of thyroid autoantibodies in relation to BPA quartiles.

Table 1 shows the clinical characteristics of the study population. The age range was between 15–98 years, with a mean age of 54.6 ± 21.5 years. The mean BMI was 23.3 ± 4.6. The proportion of subjects positive for TgAb, TPOAb and TRAb was 11.1%, 14.9% and 1.9%, respectively. With regard to BPA, 51.9% had serum BPA levels exceeding the detection limit of the assay (0.3). Figure 1 shows the percent positive rate of thyroid autoantibodies in relation to BPA quartiles. There was a significantly increasing trend for subjects with TgAb (*p* < 0.05) and TPOAb (*p* < 0.001) positivity as BPA quartiles increased, particularly in the highest quartile. In contrast, no relationship between BPA quartiles and TRAb was found. In logistic regression analysis, age, gender and BPA quartiles were determinants of TgAb or TPOAb positivity, whereas BMI was not an independent predictor (Table 2). In addition, the association between BPA quartiles and TPOAb positivity was consistent in both men and women (Table 3). In contrast, no relationship between BPA quartiles and TgAb was found in either sex (Table 4).

## 4. Discussion

All humans are now exposed to synthetic pollutants in their food, drinking water and in the air, as well as in the ordinary things they use in everyday life. Antibodies against these xenobiotics have been demonstrated in a number of studies [11,12]. There have been studies showing that BPA acts as a thyroid receptor antagonist [13] and can increase serum free T4 [6]. In the present study, serum BPA was found to be associated with certain aspects of thyroid autoimmunity, in that there was a relationship between serum BPA and TPOAb or TgAb but not TRAb. To our knowledge, this is the first report demonstrating the relationship between BPA and thyroid autoimmunity.

A causal relationship leading from BPA exposure to TPOAb positivity and subsequent autoimmune thyroid disease could not be readily determined in the present study. However, BPA may enhance autoimmunity through a number of mechanisms. For examples, BPA has higher potency than estradiol in the inhibition of monocyte-chemoattractat protein in a tumor cell line [14] as well as the adherence of macrophage [15]. Moreover, BPA can affect T-cell proliferation and Th1/Th2 polarization [16,17]. With regard to antibody production, mice fed with BPA have increased production of IgA and IgG2a [18]. Furthermore, it is noteworthy that prenatal BPA exposure may reduce TSH among newborn girls, especially among iodine deficient versus sufficient mothers, and particularly when exposure occurs later in gestation [19] which might indirectly affect the thyroid-related immunogenity. On the other hand, the observed relationship could be due to certain underlying confounders affecting both, or may be the result of reverse causation leading from autoimmune thyroid disease to altered BPA metabolism and hence increased serum BPA. Nevertheless, it has been shown in a recent study that removal of mercury-containing dental amalgam results in reduced serum TgAb and TPOAb [20]. Although mercury in dental amalgam is thought to be a causative factor of elevated serum TgAb and TPOAb, it is also known that dental amalgam is a potential source of human BPA exposure [21]. It is therefore likely that the removal of mercury-containing dental amalgam also reduces BPA exposure of the subjects. On the other hand, reverse causation, with thyroid autoimmunity increasing serum BPA levels, was less likely in the present study, as all subjects included had normal TSH and free T4.

Thyroid autoimmunity results from an interaction between genetic and non-genetic factors. A number of genes have been identified as susceptibility genes for autoimmune thyroid disease, including both major histocompatibility complex (MHC)- and non-MHC-related. Probable environmental factors include pregnancy, smoking, and iodine and selenium intake. Xenobiotics such as substances in tobacco smoke [22] and silica [23] have been associated with autoimmine diseases. With regard to endocrine disruptors, at least part of the effect of smoking can be attributed to hydroxypyridine and benzopyrene. The ubiquitous presence of endocrine disruptors in modern society may contribute to the increased incidence of autoimmune thyroid disease [24]. Exposure to thyroid disruptors in patients with vitiligo has been associated with thyroid hormone autoantibodies [25].

There are a number of limitations of the present study. As mentioned previously, causal inference cannot be readily obtained due to the cross-sectional design of the present study. With regard to BPA, the ELISA method used possesses lower sensitivity compared with liquid chromatography-mass spectrometry (LC-MS) technique [26,27]. Moreover, the serum levels of BPA found in the present study might underestimate the actual body burden of BPA in the population, and this would be expected to affect the estimated strength of the association toward the null. Moreover, the utilization of serum rather than urinary BPA may not entirely reflect daily BPA exposure since its circulatory half-life is relatively short. It has been shown that BPA level in the urine is 3 to 250 times higher than in the serum, with a mean of 42-fold [28]. In the same study, however, it was also demonstrated that both urine BPA and circulatory BPA levels are highly dependent on the timing of urine and blood collection after a meal and the BPA load in the meal. The influence of BPA exposure observed in the present study should therefore be limited only to that of a higher load of BPA rather than BPA exposure in general. On the other hand, serum rather than urinary BPA is more directly related to BPA concentrations in various tissues, where it exerts biological effects through associated receptors. Assessing serum BPA in this regard can provide additional information and should not be entirely ignored [29]. However, there is still debate with regard to the true levels of serum BPA in the general population and their biological significance. Serum BPA varies according to assay methods and the collection and handling of samples, as well as study design [30]. Serum BPA in the nanomolar range, found in some studies, is high enough to elicit biological effects through estrogen receptors [31]. On the other hand, serum BPA in the picomolar range, also found in certain studies, is likely to be too low to be biologically relevant [28].

## 5. Conclusions

BPA was independently associated with TPOAb positivity in both men and women. The finding may suggest a role of BPA exposure in autoimmune thyroid disease. However, its mechanism related to TPOAb positivity, and probable subsequent autoimmune thyroid disease, warrants further investigation.

## Figures and Tables

**Table 1:** Baseline characteristics of the study population.

Characteristics	Women (1175)	Men (1186)	*p* Value	Total (*n* = 2361)
Age (years)	58 (15–98)	57 (15–94)	0.614	57 (15–98)
BMI (kg/m^2^)	23.4 (12.7–44.8)	22.3 (13.6–43.3)	<0.001	22.7 (12.7–44.8)
Serum BPA (ng/mL)	0.30 (0.0–41.5)	0.34 (0.0–66.9)	0.009	0.32 (0.0–66.9)
Serum TgAb (IU/mL)	16.7 (10–4000)	15.0 (10–4000)	<0.001	15.7 (10–4000)
Serum TPOAb (IU/mL)	12.8 (5–6038)	12.9 (5–1447)	0.804	12.9 (5–6038)
Serum TRAb (IU/L)	0.32 (0.3–6.52)	0.37 (0.3–6.54)	0.025	0.34 (0.3–6.54)

Data are median, with range in parentheses. TgAb, antithyroglobulin antibody; TPOAb, antithyroperoxidase antibody; TRAb, antithyrotrophin receptor antibody; BMI, body mass index.

**Table 2:** Logistic regression analyses: predictors for TgAb or TPOAb positive.

Variables	TgAb Positive			TPOAb Positive		
	OR	95% CI	*p* Value	OR	95% CI	*p* Value
Age	1.01	1.00–1.01	0.036	1.02	1.01–1.02	<0.001
Male	0.35	0.26–0.46	<0.001	0.67	0.53–0.84	0.001
BMI	1.03	0.99–1.05	0.068	1.01	0.98–1.03	0.542
BPA quartile	1.13	1.00–1.27	0.049	1.34	1.21–1.50	<0.001

TgAb, antithyroglobulin antibody; TPOAb, antithyroperoxidase antibody; OR, odds ratio; CI, confidence interval; BMI, body mass index; BPA, bisphenol A.

**Table 3:** Logistic regression analyses: predictors for TPOAb positive by sex.

Variables	Female			Male		
	OR	95% CI	*p* Value	OR	95% CI	*p* Value
Age	1.01	1.01–1.02	<0.001	1.02	1.01–1.03	<0.001
BMI	1.00	0.97–1.03	0.880	1.03	0.98–1.07	0.226
BPA quartile	1.29	1.12–1.49	<0.001	1.42	1.20–1.68	<0.001

TPOAb, antithyroperoxidase antibody; OR, odds ratio; CI, confidence interval; BMI, body mass index; BPA, bisphenol A.

**Table 4:** Logistic regression analyses: predictors for TgAb positive by sex.

Variables	Female			Male		
	OR	95% CI	*p* Value	OR	95% CI	*p* Value
Age	1.00	0.99–1.01	0.795	1.02	1.01–1.04	0.001
BMI	1.01	0.98–1.04	0.576	1.08	1.02–1.14	0.005
BPA quartile	1.11	0.96–1.28	0.147	1.15	0.93–1.44	0.192

TgAb, antithyroglobulin antibody; OR, odds ratio; CI, confidence interval; BMI, body mass index; BPA, bisphenol A.
